# GPU-accelerated real-time reconstruction in Python of three-dimensional datasets from structured illumination microscopy with hexagonal patterns

**DOI:** 10.1098/rsta.2020.0162

**Published:** 2021-06-14

**Authors:** Hai Gong, Wenjun Guo, Mark A. A. Neil

**Affiliations:** Department of Physics, Blackett Laboratory, Imperial College, Prince Consort Road, London SW7 2AZ, UK

**Keywords:** structured illumination microscopy, light-sheet microscopy, super-resolution microscopy, fluorescence microscopy, reconstruction algorithm, GPU processing

## Abstract

We present a structured illumination microscopy system that projects a hexagonal pattern by the interference among three coherent beams, suitable for implementation in a light-sheet geometry. Seven images acquired as the illumination pattern is shifted laterally can be processed to produce a super-resolved image that surpasses the diffraction-limited resolution by a factor of over 2 in an exemplar light-sheet arrangement. Three methods of processing data are discussed depending on whether the raw images are available in groups of seven, individually in a stream or as a larger batch representing a three-dimensional stack. We show that imaging axially moving samples can introduce artefacts, visible as fine structures in the processed images. However, these artefacts are easily removed by a filtering operation carried out as part of the batch processing algorithm for three-dimensional stacks. The reconstruction algorithms implemented in Python include specific optimizations for calculation on a graphics processing unit and we demonstrate its operation on experimental data of static objects and on simulated data of moving objects. We show that the software can process over 239 input raw frames per second at 512 × 512 pixels, generating over 34 super-resolved frames per second at 1024 × 1024 pixels.

This article is part of the Theo Murphy meeting issue ‘Super-resolution structured illumination microscopy (part 1)’.

## 1. Introduction

Since being proposed two decades ago [1,2], structured illumination microscopy (SIM) and its derivatives have become one prevailing branch of super-resolution microscopy (SRM) techniques that surpass the Abbe limit in optical resolution determined by diffraction at approximately 200 nm laterally and 600 nm axially. Compared with some other SRM modalities that can achieve sub-100 nm to even sub-10 nm resolutions, such as those based on point spread function (PSF) engineering or single molecule localization [3–5], linear SIM techniques are still diffraction-limited and typically can only offer up to two-fold resolution improvement over conventional light microscopy. However, as a wide-field technique that requires only a few (typically 9–15) raw images to reconstruct a super-resolved image, SIM can acquire data at relatively high rates (up to 100 frames s^−1^) while imposing a relatively low light dose on the sample (less than 1 W cm^−2^ [4]). SIM techniques are also compatible with standard fluorophores and can be spectrally multiplexed with relative ease. These merits make SIM an attractive approach to imaging fluorescently labelled biological samples, including live cells, in multicolour with a high throughput and reduced photo-chemical effects [6]. Notably, by exploiting the nonlinear response of the certain fluorophores through either saturation or photoswitching, spatial frequency components beyond the limit set by the objective can be generated in the illumination pattern, making it theoretically possible to achieve unlimited resolution improvement [7–10]. However, as well as requiring higher illumination powers and/or specialized fluorophores, such nonlinear SIM methods require significantly more raw images for data reconstruction, compromising both acquisition speed and phototoxicity.

In SIM, the sample under investigation is illuminated with a structured pattern, often generated by the interference of two or more laser beams. By mixing between the spatial frequencies of the illumination pattern and those of the sample, the high-spatial-frequency components in the sample that are otherwise unresolvable by the detection microscope objective are shifted back into the passband of the objective. In the typically employed epi-fluorescence set-up, where the same objective lens is used for both illumination and detection, the achievable spatial frequency of the illumination pattern can at most be roughly the same as the maximum spatial frequency detectable by the objective lens, hence limiting the resolution improvement to a factor of 2. The mix of spatial frequencies measured in a single image with SIM is, therefore, a combination of those of the illumination and those of the sample. In order to determine just the sample contribution, the illumination is changed while recording multiple images and then these are post-processed computationally to reveal the super-resolved sample image.

In its simplest form, the illumination pattern used in SIM exhibits a sinusoidal intensity variation along one direction in the lateral sample plane, formed by the interference between two coherent laser beams. This includes three spatial frequency components in the illumination, the contribution from which can be deduced from images with the illumination pattern shifted to three different positions, usually a phase of 2π/3 apart, thus improving the resolution in the direction of the projected fringe spatial frequency vector. For a near isotropic resolution improvement in the lateral plane, the pattern needs to be rotated to two more directions with the same phase shifting repeated for each direction, as such requiring a total of nine raw images for super-resolving the data. This implementation is often referred to as 2D-SIM, as it improves only the lateral resolution but not the axial one [1,2]. It is also possible to use more than two interfering beams for generating the illumination pattern. For example, an illumination pattern with both lateral and axial sinusoidal intensity variations can be achieved with three mutually coherent laser beams. Such a pattern is employed in 3D-SIM as it improves both the lateral and the axial resolutions, albeit requiring more raw images per lateral plane than in the 2D-SIM case (typically 15 versus 9) and potentially causing more prominent photobleaching/phototoxicity if a large number of lateral planes were to be imaged for producing a volumetric dataset [11]. Another example is the use of three or more beams to generate an illumination pattern with intensity variations along multiple lateral directions [12,13]. This type of pattern makes it possible to achieve a more isotropic lateral resolution improvement without the need of rotating the pattern [14,15]. For a detailed discussion on how the various illumination patterns affect the performance of SIM systems, refer to [16].

Besides the hardware configuration, the performance of an SIM system depends heavily on the post-processing algorithms that convert the acquired raw data into super-resolved images [17]. The post-processing procedure generally involves two steps, namely parameter estimation and image reconstruction. The parameter estimation step aims to retrieve the relevant parameters about the illumination patterns from the raw images of the sample under investigation or of phantom data such as fluorescent beads. With the retrieved parameters, in the image reconstruction step, the down-modulated high-spatial-frequency components can be isolated and shifted back to their true positions in the Fourier space. The resulting enlarged Fourier spectrum is then further filtered (often with a Wiener filter) for deconvolution as well as artefact suppression before undergoing an inverse Fourier transform to yield the super-resolved reconstructed image. Accordingly, various algorithms have been developed for these tasks, and, especially in recent years, the focuses have been on accurately estimating the relevant parameters from raw images with extremely low SNR, suppressing reconstruction artefacts, and increasing the overall speed of the whole post-processing procedure [11,18–28]. Some open-source software packages have also been made available [29–31], including the recent fairSIM-VIGOR that enables real-time SIM reconstruction at video frame rates [32], which offer promising performance and make it easier for end users to process SIM data. However, it should be noted that these software packages are designed for processing generic SIM data from commercial systems or from bespoke systems using simple linear grids for illumination; for processing data with a different SIM pattern, while the data may be transformed so as to be compatible with the generic software packages, it is worth developing a new package from scratch for a minimal codebase and optimized performance.

In SIM, it is the changes in the image as the structured illumination pattern is altered that enable both the measurement of the structured illumination pattern's parameters and the subsequent reconstruction of super-resolved images. It is, therefore, beneficial that either the sample is thin or that, alternatively, only the in-focus parts of the sample are illuminated by performing SIM with light-sheet illumination. To this end, schemes based on single or multiple Bessel beams [33,34] and lattice light sheet [10,35,36] have been used to improve the axial sectioning of light-sheet microscopes. However, it has also been demonstrated that the interference between two counterpropagating coherent light sheets [37] can be exploited to increase the lateral resolution by more than a factor of two as the illumination is generated outside the constraints of the numerical aperture of the imaging objective. As well as increasing contrast by limiting illumination to just the in-focus parts of the sample, light-sheet-based SIM reduces the overall light dose to the sample and specifically photobleaching and phototoxicity outside the illuminated plane.

**Figure 1.**
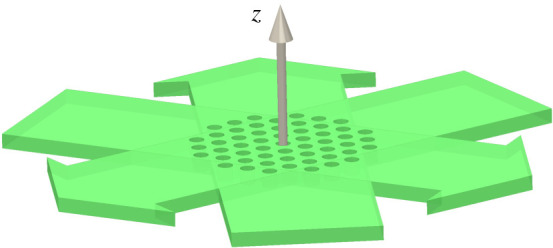
The design of a light sheet SIM system with hexagonal patterned illumination. Three light sheets (green) are coincident at mutual angles of 120° in the focal plane of the microscope to form a hexagonal structured light sheet. Cells flowing through the light sheet in the +*z*-direction emit fluorescence that is collected in the +*z*-direction by an objective lens and imaged onto a camera. (Online version in colour.)

In this paper, we present hexSIM, an implementation for performing 2D-SIM with hexagonal illumination patterns that is compatible with lab-on-a-chip light-sheet implementations such as described recently [38]. A proposed design based on this implementation is presented in figure 1. A structured light sheet with the required hexagonal illumination pattern may be formed with three mutually coherent light sheets overlaying at the focal plane of the detection objective lens. Phase shifting the pattern may be achieved by simply altering the phases of two of the three light sheets. This arrangement does not suffer from the disadvantages that it would have in an epi-illumination set-up, as identified in [16], namely a limited resolution enhancement and reduced modulation amplitude in the illumination pattern. As the illumination is outside the objective NA, we expect the full two-fold improvement in resolution, and with illumination polarized along the objective optic axis, we see a doubling of the modulation amplitude in the illumination pattern compared to the normal azimuthal polarization required in epi-illumination, as described in the supplementary material. Combined with an on-chip microfluidic channel, within which samples can be delivered across the structured light sheet along the axial direction of the detection objective lens, our method should serve as a promising means for high-throughput volumetric imaging with real-time super-resolved outputs.

We also present a post-processing algorithm tailored for hexSIM, which requires only seven raw images for reconstructing a super-resolved frame. We demonstrate the operation of the algorithm on experimental data from an epi-illuminated set-up using a ferroelectric liquid crystal spatial light modulator (FLCSLM) to generate the required hexagonal illumination patterns, showing that hexSIM with epi-illumination is capable of achieving an up-to-1.87-fold near isotropic improvement in the lateral resolution. We also demonstrate with simulated data that the custom algorithm written in Python can be GPU-accelerated to perform real-time image reconstruction on standard personal computers with raw images acquired at a frame rate of greater than 100 fps. We investigate by simulation the potential for the system to generate artefacts while imaging samples that are moving through the focal plane as a 3D stack is produced and demonstrate a simple processing solution that removes those artefacts.

## 2. Methods

### (a) Structured illumination microscopy with two-dimensional hexagonal illumination patterns

Our SIM methodology, hexSIM, employs two-dimensional hexagonal illumination patterns for improving lateral resolution. This is illustrated for a system with epi-illumination: by focusing three mutually coherent beams to the pupil plane of the illumination objective lens (figure 1*a*), a hexagonal intensity illumination pattern like that illustrated in figure 1*b* is formed at the sample plane. Also shown in figure 1*a* are the seven spatial frequency components in the illumination intensity pattern plotted again relative to the objective pupil. It can be seen here that when the illuminating beams are positioned at the edge of the pupil (the coherent cut-off) then the spatial frequency is only at a factor of $\sqrt{3}$ larger than the coherent cut-off. Combined with the fact that the incoherent cut-off spatial frequency seen when imaging the light back through the objective is twice the coherent one, this is what results in a limit of $1+\sqrt{3}/2=1.87$ improvement in resolution in epi-illumination.

Unmixing the seven spatial frequency components generated by this type of illumination pattern requires only seven raw images illuminated with seven appropriately phase-shifted patterns (see electronic supplementary material, S1 for a detailed mathematical description), two fewer than the nine raw images required by conventional 2D-SIM, making it attractive for high-speed imaging. Another attractive feature is that the illumination pattern (or its generating beams) does not need to be rotated in order to achieve near isotropic resolution improvement. In fact, altering the phase of just two of the beams is enough to move the pattern laterally and arbitrarily in the sample plane.

While with an epi-illumination set-up, the lateral resolution improvement of hexSIM is limited to only approximately 1.87, this limitation can be overcome by delivering the illumination beams as light sheets (figure 1*c*), where the illumination numerical aperture (NA) is now decoupled from the detection NA. We envisage that such a light-sheet illumination set-up may be achieved with a photonic-chip-based system similar to that reported in [38], where three mutually coherent and overlaying light sheets are generated with on-chip optics, and the hexagonal interference pattern can be shifted laterally by shifting the phases of two of the three light sheets. As the light sheet illumination is orthogonal to the optic axis of the imaging objective ($\theta =90^{\circ }$), so the effective illumination NA=nsin⁡θ=n, the refractive index in the sample. So taking the objective used in [38] as an example (NA = 1.1), then with a sample refractive index of *n* = 1.33, equivalent to water, we would expect a resolution enhancement of $\left(2\text{NA}+\sqrt{3}n\right)/2\text{NA}=2.05$. With the higher refractive index sample media used in [38], this would rise to 2.15.

**Figure 2.**
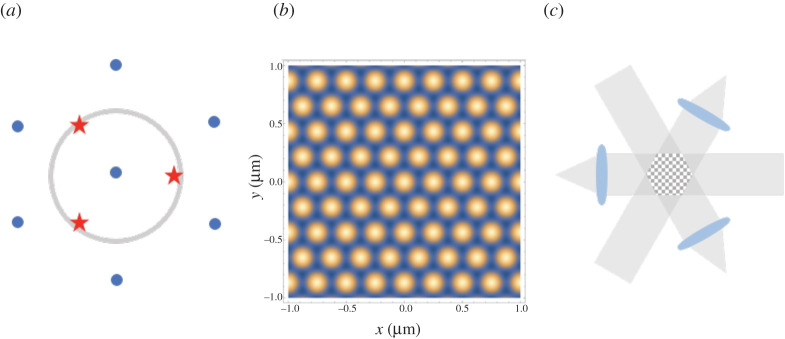
Illumination pattern design for hexSIM. (*a*) The locations of the three illumination beams (red stars) and the seven spatial frequencies from the illumination pattern (blue spots) relative to the illumination pupil (grey circle). (*b*) Simulated illumination pattern for hexSIM. (*c*) Potential light-sheet-based illumination scheme for hexSIM. (Online version in colour.)

A further complication of the epi-illumination set-up is that it usually requires the polarization of the illumination beams to be arranged azimuthally with respect to the pupil and in this arrangement, the illumination intensity is not actually as shown in figure 2*b* but rather its reversed contrast, where as a result, the amplitude of the higher spatial frequency components is halved. In light-sheet mode, the equivalent would be to arrange for the polarization of the input beams to be in the plane orthogonal to the optic axis of the imaging objective (in-plane polarization). Arranging the polarization to be oriented along the optic axis (axial polarization) does, however, result in the pattern shown in figure 2*b*, and maximizes the amplitudes of the higher spatial frequency components. The equivalent radial polarization in epi-illumination is usually avoided as it results in a mix of positive and negative contrast hexagonal patterns and often then a much-reduced overall contrast in the illumination. These polarization issues are discussed further in electronic supplementary material, S2.

To experimentally demonstrate the capabilities of our hexSIM methodology and the tailored post-processing algorithm introduced below, we built an FLCSLM-based epi-illumination set-up with a 40 × 0.75 NA air objective and azimuthally polarized excitation. Imaging experiments were performed on samples with fluorescent microspheres and on samples with fluorescently labelled fixed cells. Details of the system and of the samples can be found in electronic supplementary material, S3 and S4, respectively.

### (b) Post-processing algorithm for hexSIM

Referring to the principle as mathematically described in electronic supplementary materials, S1, hexSIM can reduce the required raw image number to 7 to produce a full two-dimensionally super-resolved image. As hexSIM and conventional two-beam 2D-SIM with three orientations share the same spatial frequency components in their illumination patterns, their ultimate reconstructed image characteristics will be similar and in addition, the seven acquired raw hexSIM images can be converted equivalently to the nine images that are usually acquired by a conventional two-beam SIM, with three phase shifts at three orientations. Processing the seven hexSIM raw images can, therefore, be achieved by first decomposing them linearly into nine images from the two-beam SIM system and then processing them using conventional two-beam SIM reconstruction algorithms such as fairSIM [31], and indeed, we follow this procedure for comparison. Here, however, we have developed an algorithm specifically optimized for our hexSIM system that can process the seven raw images directly.

**Figure 3.**
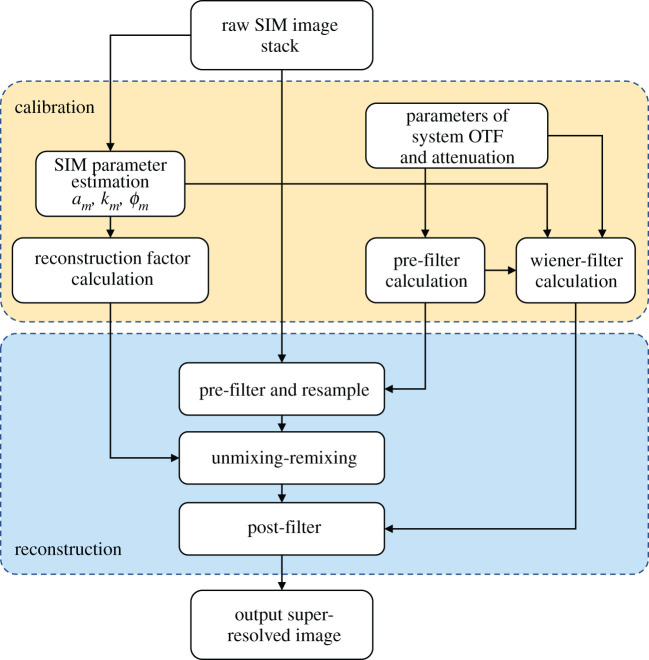
The flowchart of hexSIM analysis process. The calibration phase measures the parameters of the applied structured illumination from a block of seven acquired raw input images of the sample. Along with other user supplied reconstruction parameters, it then calculates constant arrays that are used in the reconstruction phase. The reconstruction phase consists of three stages as shown taking the input data, pre-filtering and up-sampling it, unmixing and remixing the different carrier bands in the image and finally post-filtering to produce an output super-resolved image. (Online version in colour.)

The generic scheme of processing our hexSIM data is described in figure 3. It composes two parts: calibration and reconstruction. The calibration stage characterizes the illumination patterns projected onto the sample and then pre-calculates look-up tables of the various filters and other parameter arrays used in the reconstruction phase where the final super-resolved images are produced. In general, this procedure is similar to that used in other approaches such as described in [2,31], but in this case, we are optimizing our implementation to concentrate on processing hexSIM data and the reconstruction phase in particular to make sure that this can run at a faster rate than a target of 100 fps of raw frames of size 512 × 512 pixels. To test the algorithm performance we also simulated, with 3D point clouds, raw hexSIM image stacks as would be acquired with a system with light-sheet illumination and automated sample delivery across the structured light sheet along the axial direction of the detection objective. The method used for simulating the data is provided in electronic supplementary material, S5.

#### (i) Calibration

The inputs are a minimum of seven raw images from the above-described illumination with the sequential seven phase-shifted hexagonal patterns. Additional parameters of the system optical transfer function (OTF) are needed to set the pre-filter including an attenuation factor applied to adjust the low spatial frequencies of the OTF in order to supress out-of-focus signals. These parameters can be either calculated from the optical set-up, provided as user inputs or derived from the calibration measurement. The pre-filter is used in the first step of reconstruction to condition and resample the input data, as well as in the construction of the final Wiener filter.

To process the raw images correctly, the calibration stage estimates the parameters of the illumination patterns, often from the raw data itself or images of phantom data such as fluorescent microspheres. The parameters that the calibration stage aims to find are the orientation and scale *k_m_*, modulation amplitude $\alpha_{m}$ and spatial phase $\phi_{m}$ of the applied illumination pattern's spatial frequency components. These are found by analysing the images using cross-correlation techniques [39]. These estimated parameters are then used to calculate the reconstruction factors *Y_n_* according to electronic supplementary materials, equation (S16). The unmixing–remixing process in the reconstruction stage involves the isolation of the various bands associated with the illumination pattern spatial frequency components, shifting them to new positions in Fourier space and hence increasing resolution. This process is actually achieved by multiplying the pre-filtered and resampled raw input images in the spatial domain by a set of reconstruction factors *Y_n_* arrays as described in electronic supplementary material, equation (S15), to produce an intermediate image stack *I*_int_.

The calibration stage also uses the calculated pre-filter and the measured SIM parameters to calculate a Wiener filter [11] that is used at the final post-filtering stage. The Wiener filter, rather than being designed to produce a flat frequency response, includes a regularization term that attenuates the output towards higher spatial frequencies so that the output images appear as natural as possible with minimal ringing artefacts. The design of the Wiener filter also requires a user parameter to characterize the expected signal and noise level in the data such that it avoids amplifying noise in parts of the image spectrum where the OTF and, therefore, the signal is small. The final post-filter stage combines the intermediate images *I*_int_ and applies the combined Wiener filter and output regularization so as to produce a super-resolved image.

#### (ii) Reconstruction

**Figure 4.**
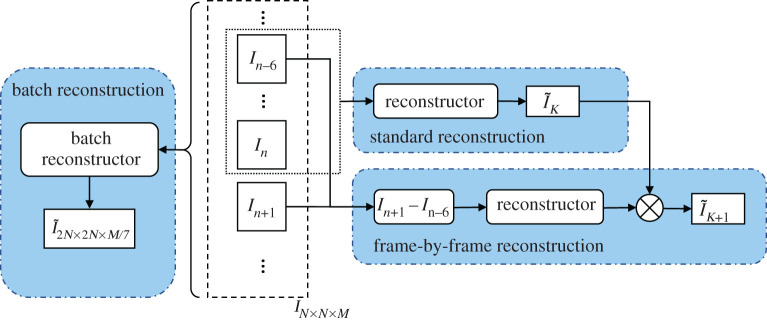
The strategies of different reconstruction process: standard reconstruction, frame-by-frame reconstruction and batch reconstruction. (Online version in colour.)

After the calibration phase, the pattern parameters have been estimated and used to generate a set of look-up tables to use in subsequent reconstructions. This calibration state is not so time crucial as a single set of calibration parameters and look-up tables can be used for multiple reconstructions. Here, we consider the implementation of three methods to process the raw data for different situations to meet the target of real-time processing: standard reconstruction, frame-by-frame reconstruction and batch reconstruction, as shown in figure 4. All three methods use the same pre-filter, unmixing–remixing, post-filter methodology and look-up tables described in figure 3 but operate on differing numbers of input frames.

Standard reconstruction is performed on a single set of seven N×N pixel images ($I_{n-6}$ … $I_{n}$) of an object with the seven required illumination patterns. It produces a single 2D super-resolved image ${\tilde{I}}_{k}$ of size 2N×2N and is suitable for isolated sets of unrelated SIM images, e.g. where a 2D super-resolved snapshot is required.

The frame-by-frame reconstruction is particularly designed for the reconstruction of streamed data. The light-sheet SIM system has been designed to produce 3D datasets by imaging cells as they flow through the object plane of the microscope. As such, the hardware produces a stream of images cycling through both the height *z* and the seven illumination patterns. After acquiring the (n+1)th frame in this sequence, it is thus possible to combine the seven frames (n−5) to (n+1) to generate a new super-resolved image for each new raw image ${\tilde{I}}_{k+1}$ in the sequence using the standard reconstruction. However, as the reconstruction process is a purely linear process, all that needs to be done is processing the difference between frame (n−6) and frame (n+1). The previous processed frames will have been stored internally and only this difference is processed to produce a new output super-resolved frame.

**Figure 5.**
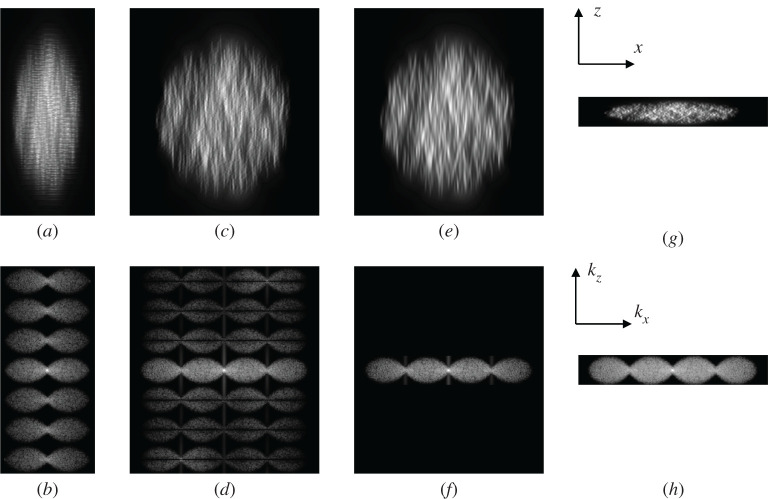
Results of processing a stack of 280 images of a simulated moving object (*a*) projection along the *x*-axis of the super-resolved stack image showing reconstruction artefacts (*c*) as a result of the object motion. (*b*) Slice in *yz* plane of the Fourier spectrum of the 3D image shown in (*a*). (*d*) Slice in *yz* plane of the Fourier spectrum of the reconstructed 3D image shown in (*c*). (*f*) Spectrum in (*d*) filtered to just the zero band in *z* and the spatial domain reconstruction of this showing reduced artefacts (*e*). (*g*,*h*) The results of the batch reconstruction method that perform the filtering in *z* as part of the unmixing–remixing process and results in a 7*x* reduction in data to just 40 planes and a close to Nyquist sampled output image. Videos of the data represented in (*a*,*c*,*e*,*g*) as the *xy*-plane is moved through *z* are given in the electronic supplementary material, Movies S1–S4, respectively. The data shown in (*a*) are also filtered in *z* in the same way as that shown in (*e*) in order to generate a wide-field *z*-stack and are shown in electronic supplementary material, Movie S5. (Online version in colour.)

Batch reconstruction is implemented to handle segmented 3D datasets. The cell is illuminated by the structured light-sheet pattern as it flows through the fluidic channel. A 3D stack of images of the cell is acquired as the cell flows in *z* and the hexagonal pattern is simultaneously and repeatedly shifted through its seven positions. Essentially, the further complication of imaging a moving object under SIM illumination gives rise to artefacts in the final processed super-resolved image, as both the illumination and the object are changing every time a new image in the stack is recorded. This is highlighted by the images shown in figure 5. Here, a raw dataset consisting of 280 frames of 128 × 128 pixels is simulated of 1000-point sources randomly distributed over a 10 µm diameter spherical region. Images are captured as the set of points moves through the focus of the microscope while presenting the seven hexagonal structured illumination patterns in turn. Figure 5*a* shows the raw simulated dataset summed along the *y*-axis. The changing illumination patterns can clearly be seen in the *z*-direction as well as the slower variation of the intensity as the object moves through focus, a result of the axial resolution of the imaging objective lens and the axial extent of the light-sheet illumination. As a further illustration of this dataset, the Fourier spectrum of the data (a slice through the *k_x_k_z_*-plane) is shown in figure 5*b*. A classic section through the 3D OTF of the imaging objective is seen along with replications of this along the *k_z_* axis due to the changing illumination patterns. Note that the rate that the object moves through *z* has been chosen such that it is adequately sampled so that these higher frequency bands do not overlap. Figure 5*c* shows the image as it would be reconstructed using the frame-by-frame method. Here, fine structure can still be seen in the *z*-direction in the image, which is clearly illustrated in the frequency spectrum shown in figure 5*d*. The zero-order spectrum is as would be expected from the SIM reconstruction, including a doubling in lateral resolution, but it is clear that there is a set of attenuated higher side-lobes in the *k_z_*-direction that are responsible for the finer structure seen in the *z*-direction in figure 5*c*.

If we apply a further filtering step and block these higher side-lobes altogether, as shown in figure 5*f*, then the resulting real image shown in figure 5*e* no longer shows the artefacts in the *z*-direction. The results in figure 5*e,f* show that we can improve on the frame-by-frame processed stack when looking at 3D datasets. Indeed, they also indicate that we can reduce the data that we store from the final processed image stack by a factor of 7 as only 1/7th of the spatial frequency spectrum contains any information. However, in arriving at this result, we have included a further filtering step in the pipeline. Looking back at the frequency spectrum in figure 5*d*, we can see that its characteristics show zeros at repetitions of the SIM pattern repetition frequency that are due to the summation over 7 intermediate image frames as part of the unmixing–remixing process, resulting in an effective apodization in the frequency domain by a *sinc*-function in *k_z_*. It should, therefore, be possible to perform an improved version of this summation step by avoiding the summation over seven intermediate images in the mixing–unmixing stage and instead directly selecting just the zero-order terms from the Fourier spectrum in *k_z_* in the final post-filtering stage. This is illustrated by the final image spectrum shown in figure 5*h* with the reconstructed image shown in figure 5*g*. Clearly, the artefacts in *z* have been removed and simultaneously the output data has still been sampled sufficiently while removing redundant data. The output data here are only 256 × 256 × 40 pixels, i.e. a sevenfold reduction in required data storage. Provided the original data was adequately sampled in *z* then this final processed dataset can be arbitrarily resampled in the *z*-direction as desired at a later date. This is the basis of the batch reconstruction method. A whole stack $I_{N\times N\times M}$ can be processed in one go, reducing the modulation artefacts, and producing an output image stack of dimensions $I_{2N\times 2N\times M/7}^{\text{out}}$. The method also shows some speed improvements compared to the frame-by-frame method as the data are down-sampled before passing onto the post-filtering step, so a sevenfold reduction in execution time of that step can be expected.

#### (iii) Implementation in Python using the CUDA-based CuPy package

A Python version of the above-described processing scheme and methods has been written, concentrating on the reconstruction process, which is most critical for real-time data processing. In general, the steps required use fast Fourier transform (FFT) routines to convert between real and frequency spaces, and then array operations such as multiplications and additions to implement the filtering operations and mixing–unmixing. These highly parallel operations are well supported by the Python language and packages such as NumPy [40] and PyFFTW [41]. However, with larger datasets, it is desirable to be able to use the GPU-based processing as the hardware is optimized well to process these standard and highly parallel array operations. For this, we used the CuPy [42] package in Python. This has the advantage that its interface is designed to follow that of NumPy code such that the code is directly portable between the two. The only difference is that if the data are stored in a cupy.ndarray instead of a numpy.ndarray then it will be stored in GPU memory and processed by routines running on the GPU. The restriction is that CuPy only supports Nvidia GPU chips as it is based on CUDA. There is an equivalent approach, ClPy [43], being developed based on OpenCL that can support a much wider range of GPUs, though this is not currently so well developed and does not currently provide all the routines required for our calculations. We also implemented some of the routines using the OpenCV package [44], which is capable of accelerated performance from within Python on CPUs and GPUs using both OpenCL and CUDA. However, as it does not have routines for efficiently handling three-dimensional arrays, it could not be used effectively for the batch reconstruction method, so the results are omitted here.

The complexity and execution times of the various reconstruction methods are highlighted below in the Results and discussion section. Here, we compare the performance of our code on datasets similar to those shown in figure 5 but with a stack-size of 512 × 512 × 280 pixels. All calculations were carried out at 32-bit single precision floating point mainly as this is preferred for floating point calculations on most lower cost GPUs but also in order to reduce the data size, both in terms of absolute storage memory requirements and in terms of the amount of data that had to be moved between CPU and GPU for processing. To this end, we also used so-called realFFT functions as our data are real in the spatial domain and, therefore, Hermitian-symmetric in the frequency domain. This has the benefit of both reduced computational overhead and half the memory storage requirements over fully complex FFT routines.

### (c) Reference computer systems for performance measurement

Three computer systems were used to evaluate the performance of our algorithms. These included a workstation class machine with external high-performance gaming GPU on PCI-express, a laptop with integrated external graphics chip and an embedded system with integrated graphics. The details of the three systems used are given in table 1. The systems were chosen as they included a range of different CPUs, GPUs and operating systems. All reference systems were installed with Python (v. 3.8), NumPy (v. 1.19.0), PyFFTW (v. 0.12.0) and CuPy (v. 7.6.0).

**Table 1.** The workstation, laptop and embedded reference systems used for assessing code performance. Three different configurations are chosen representing a range of hardware architectures and operating systems.

	computer model	CPU	GPU	memory	operating system
workstation	HP Z420 Workstation	Intel Xeon E5-1650 (6-core, 3.2 GHz)	NVIDIA GeForce GTX1070	32 GB (main)8 GB (GPU)	Windows 10
laptop	HP ZBook Studio G5	Intel Core i5-8400H (4-core, 2.5 GHz)	NVIDIA Quadro P1000	16 GB (main)4 GB (GPU)	Windows 10
embedded	Nvidia Jetson AGX Xavier	ARM v8.2 (8-core, 2.1 GHz)	512-Core Volta with Tensor cores	16 GB (shared)	Linux

For performance tests, the calibration and reconstruction routines were run and timed either as a single instance (calibration and batch reconstruction) or multiple times to produce an average execution time (standard and streaming reconstructions). All calculations were performed on simulated point cloud data similar to that shown in figure 5 at 512 × 512 raw image size with 1024 × 1024 output image size. The performance of the code is not expected to be dependent on the information in the input images other than their size.

## 3. Results and discussion

### (a) Structured illumination microscopy imaging from epi-fluorescence set-up

**Figure 6.**
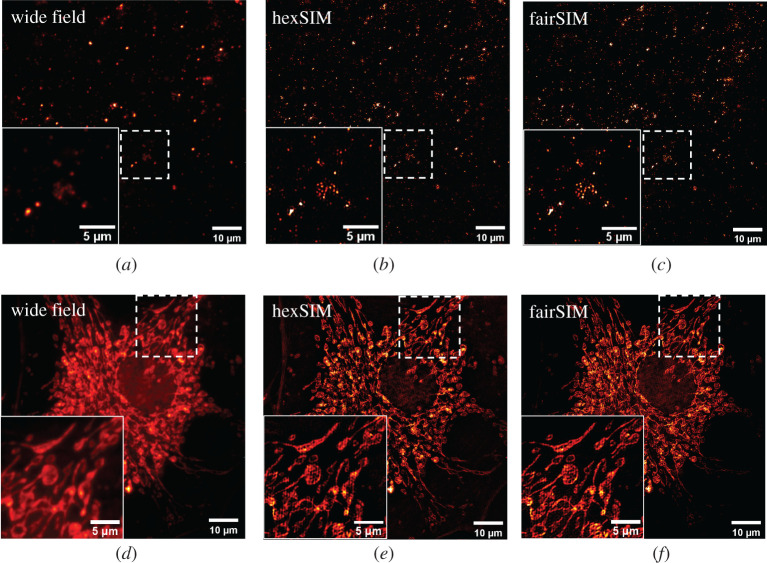
(*a–f*) Calibration of the hexSIM system with a fluorescent microspheres sample (0.1 µm TetraSpeck™ microspheres, ThermoFisher). Wide-field image (*a*), hexSIM image (*b*) and fairSIM image (*c*). From a decorrelation analysis, the estimated resolution of the wide-field image is 474 nm. For the hexSIM image and fairSIM, it is 267 nm and 259 nm, respectively. (*d–f*) Experimental results of imaging the BPAE cells (FluoCells Prepared Slide #1, ThermoFisher). Comparison of the wild-field image (*d*), hexSIM image (*e*) and fairSIM image (*f*). The estimated resolutions are 528, 296 and 266 nm from left to right. Zoomed in views of the same area are provided in the down left corner. The hexSIM reconstructed the image from a dataset of seven raw images. The fairSIM processed image is reconstructed from a linear decomposition of this dataset into nine images. (Online version in colour.)

An experimental illuminated set-up, as described in electronic supplementary material, S3, has been constructed to validate the principle and software of a hexSIM system. To evaluate the improvement of resolving power of hexSIM, we first imaged a sample of 0.1 µm fluorescent microspheres (T7279, TetraSpeck, Invitrogen), prepared as described in electronic supplementary material, S4. Figure 6*a,b* shows the wide-field image (512 × 512) and hexSIM image (1024 × 1024) of the fluorescent microspheres. Comparing the zoomed area, the microspheres in the hexSIM image are clearly separated and reconstructed with improved contrast, such that many beads not visible in the wide-field image are now clearly resolved.

The reconstruction results of our hexSIM and the open-source software fairSIM are compared. The hexSIM image in figure 6*b* is directly reconstructed from seven raw images under the hexagonal pattern illumination. This acquired dataset was also linearly decomposed to a nine-image dataset which is equivalent to that of a standard two-beam SIM with three projected spatial frequency components and three phase shifts. This transformed dataset was processed with the fairSIM ImageJ plugin to produce the super-resolved image in figure 6*c*. The hexSIM image and the fairSIM image are similar to a large extent, and both have shown a significant resolution improvement when compared with the wide-field image in figure 6*a*.

A well-established decorrelation analysis based on image partial phase autocorrelation [45] is used to estimate the resolution of the imaging system. The images are processed with Image Decorrelation Analysis ImageJ plugin provided by the authors. The calculated resolution of the wide-field image figure 6*a* is 474 nm. The resolutions of the hexSIM image and fairSIM image are found at 267 nm and 259 nm, respectively. The theoretical diffraction limit resolution of the microscope is estimated from λ/(2NA)≈390 nm for the emission wavelength *λ* = 580 nm and NA = 0.75 used here. The approximated resolution improvement of the hexSIM compared to the wide-field image system is 1.78. For the fairSIM, the improvement is slightly higher at 1.83. They are both close to the theoretical resolution improvement limit of 1.87 as described in the Methods section, though there is a room for hexSIM to improve by optimizing the parameters of the filters.

A biological sample of the BPAE cells (FluoCells slide #1, ThermoFisher) is imaged (wide field: figure 6*d*, hexSIM: figure 6*e*, fairSIM: figure 6*f*) by observing the cell mitochondria that are labelled with MitoTracker Red CMXRos. A resolution of 528 nm is estimated by decorrelation analysis for (figure 6*d*). And again, the hexSIM image in figure 6*e* shows a 1.78-fold improved resolution of 296 nm. The resolution of fairSIM image in figure 6*f* is estimated at 266 nm. In inspection of the image quality, the fairSIM image in figure 6*f* shows a lower contrast in the sparse region and appears smoother. This difference could be caused by the different attenuation strategies used for the OTF and filtering. The result from fairSIM contains more background artefacts than hexSIM. This noise could be introduced by the assumption in the dataset decomposition that the amplitudes of all the spatial frequency components are equal. These results suggest a comparable reconstruction between the hexSIM and the fairSIM processing methods.

### (b) Software performance

We have tested our code on multiple platforms with different CPU and GPU models as described in the Methods section. The settings and results are shown in table 2. Since hexSIM is targeted for being used in a high-throughput imaging platform by imaging cells as they flow through the focal plane in a microfluidic channel, the acquisition and reconstruction need to operate at a high speed to match the flow of the cells. To this end, we also list the target execution time for the different methods in order to be able to give a processing frame rate of 100 fps raw image data.

**Table 2.** Comparison of the performance of the hexSIM processing code on different platforms and methods.

method	calibration	standard reconstruction	frame-by-frame reconstruction	batch reconstruction
target reconstruction time		70 ms	10 ms	2.8 s
array size	512 × 512 × 7	512 × 512 × 7	512 × 512	512 × 512 × 280
laptop (CPU)	1.42 s	89.2 ms	26.8 ms	9.7 s
workstation (CPU)	1.25 s	80.4 ms	24.8 ms	7.6 s
embedded (CPU)	1.12 s	75.4 ms	19.2 ms	5.6 s
laptop (GPU)	0.89 s	17.6 ms	6.4 ms	1.73 s
workstation (GPU)	0.82 s	22.9 ms	5.7 ms	1.17 s
embedded (GPU)	0.64 s	30.0 ms	10.2 ms	1.98 s

For all the platforms, the standard reconstruction in CPU mode takes more than the target 70 ms for the dataset size of 512 × 512 × 7. The frame-by-frame reconstruction aimed at displaying streamed data takes more than 19.2 ms and batch reconstruction more than 5.6 s. From the results, it can be observed that the CPU-only mode on all the platforms cannot fulfil the real-time reconstruction time requirements. A CuPy-based version of hexSIM as introduced in the Methods section was deployed on all three platforms as well. The GPU enabled an acceleration of between 2 and 6.5 times on all the reconstruction methods and the processing target is reached for all three platforms for all three methods apart from the embedded system and frame-by-frame reconstruction, which misses by 0.2 ms. On the workstation, which is used with the experimental set-up used above, a processing rate of 175 fps has been achieved. For the segmental 3D reconstruction, only 1.17 s is required to produce 40 frames of super-resolved images from an input stack of 280 imaged. Even on a low-cost embedded GPU-based computer, the achieved speeds meet the targets for the standard and batch reconstruction methods. With smaller image sizes as might be produced by segmenting the field to capture single cells at 256 × 256 raw data pixels per frame, the processing speed improvements using the GPU has the potential to process two-colour datasets at 200 fps.

Though there is no real-time requirement for the calibration process, a faster version of the parameter estimation has also been implemented via partial coding with CuPy. The speed of calibration has increased over 30% on all three platforms.

Clearly, the performance of the different systems and methods is not just a function of CPU and GPU processing power. The memory required for storage and transfer between CPU and GPU are often critical in applications such as this. For example, the acquired raw data at 16 bpp (bits per pixel) for a 512 × 512 × 280 dataset are over 140 MB that must be transferred to the GPU, and with batch reconstruction, the output data size at 1024×1024×40 and 32 bpp is 160 MB that must be transferred back to CPU space. Internally, with single precision floating point calculations (32 bpp) and expansion to 1024×1024 frame size, some of the internal data structures that are produced during intermediate calculations are over 1.1 GB in size in batch mode and the limited GPU RAM available can become an issue.

**Figure 7.**
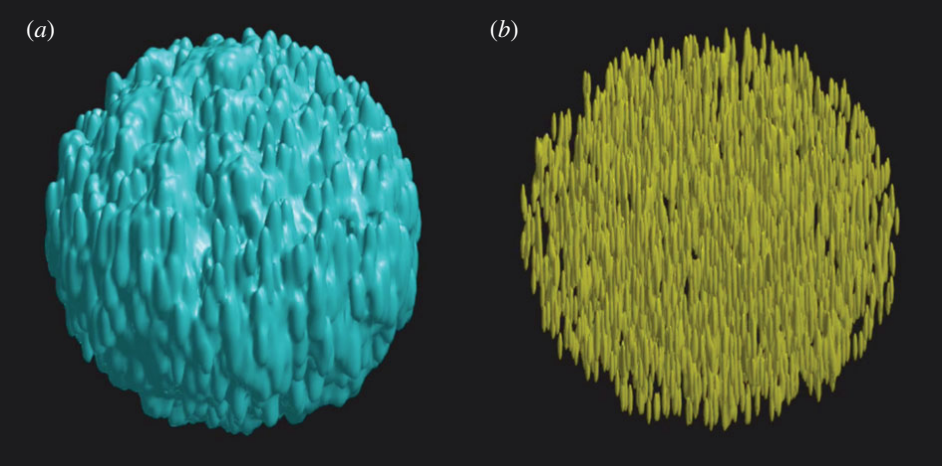
A 3D iso-surface reconstruction of a 1000 point cloud dataset distributed randomly over a 10 µm diameter sphere. (*a*) A conventional wide-field image reconstructed from the simulated data while (*b*) shows the super-resolved image reconstruction. A movie file of these data is available in the electronic supplementary material, Movie S6. (Online version in colour.)

While the batch reconstruction method is capable of removing the artefacts that arise in standard and frame-by-frame reconstructions due to the sample moving through focus as the seven different structured illumination patterns are acquired, this also points to a requirement to satisfy Nyquist sampling in the *z*-direction that is seven times that for normal wide-field imaging. For normal wide-field imaging, the sampling requirement in *z* is given by: $dz_{\text{Nyquist}}=\lambda /2(n-\sqrt{n^{2}-\text{N}{\text{A}}^{2}})$. Using the parameters λ = 525 nm, NA = 1.1 and *n* = 1.33, this gives $dz_{\text{Nyquist}}$ = 450 nm, implying that the hexSIM data should be samples at most at *dz* = 64 nm. At 100 fps frame acquisition rate, this implies a maximum speed of cells flowing through the focal plane of 6.4 µm s^−1^. In addition, with a light-sheet illumination, the resolution in *z* will be further enhanced, though with the simulations considered here and a 3 µm Gaussian width light sheet, only a 20% reduction in sampling distance is sufficient resulting in *dz* = 50 nm or 5 µm s^−1^ cell flow speed. An example of reconstruction of a dataset simulated at *dz* = 50 nm is shown in figure 7.

## 4. Conclusion

We have validated a structured illumination microscope with hexagonal patterns (hexSIM) that could be deployed on an optofluidic chip in a light-sheet mode. This system aims at real-time, high-throughput and super-resolving imaging of the cells flowing through a microfluidic channel. The post-processing algorithm serving for hexSIM has been explicitly described in this paper. Three reconstruction methods have been implemented in Python to process different datasets. For a standard reconstruction, seven phase-shifted raw images are required to produce a super-resolved image. A frame-by-frame reconstruction is developed for processing high-speed streamed data in real time. A batch reconstruction is implemented to process large 3D datasets that may contain images of whole cells. This last reconstruction method in particular has been proved to be able to negate the motion artefacts produced in the other reconstruction methods caused by the movement of the sample in *z* as a set of structured illumination patterns are projected onto the sample. Python not only provides a convenient way to develop and distribute code, but also the required core numerical methods used in the reconstruction process are well supported by open source and optimized Python packages. In particular, we demonstrate that the algorithms are further accelerated by parallel processing with the assistance of GPU by using the CuPy package in place of NumPy for these core operations.

The feasibility of this technique and the performance of the customized software were validated in a proof-of-principle set-up built in an epi-illumination configuration. In the experiments, we show a 1.78-fold increase in resolution estimated by autocorrelation of microsphere bead images that agrees with the theoretical estimate of 1.87-fold improvement. The image quality of cells reconstructed by hexSIM is comparable to the commonly used fairSIM. In terms of the post-processing speed, a significant acceleration has been observed by processing the data on a GPU. The real-time reconstruction requirement at 100 fps for a frame size of 512 × 512 pixels has been fulfilled for simulated datasets on a range of computer systems including a workstation, a laptop and an embedded computer. A frame-by-frame processing speed of 175 fps for 512 × 512 pixel frames was recorded on the workstation that incorporated a gaming GPU card. When processing in a batch mode at a batch size of 280 frames, an effective frame rate of 239 fps is achieved generating over 34 fps of super-resolved images at 512 × 512 pixel resolution. Combined with the fewer frames required by hexSIM, the fact that near isotropic lateral resolution can be achieved with just 3 fixed-position illumination beams makes the whole system promising for the realization of subdiffraction resolution in a light-sheet geometry on a microfluidic chip. It should also be straightforward to adapt hexSIM to most existing 2D-SIM systems, as it requires no change in hardware but the display of a different set of patterns on the SLM. Moreover, we believe that our hexSIM concept is compatible with the on-chip TIRF-SIM system proposed by Helle *et al*. [46] and may simplify their chip design by reducing the number of waveguides needed.

## Data accessibility

Raw data from experiments and simulations, the code for generating the simulated data and the code for post-processing the raw data can all be found at https://dx.doi.org/10.5281/zenodo.4315021.
